# Association Between Serum Albumin Levels and Acute Kidney Injury in Patients With Acute Pancreatitis: A Systematic Review and Meta-Analysis

**DOI:** 10.7759/cureus.98952

**Published:** 2025-12-11

**Authors:** Mohamed Idris, Mohammed Abdalazeem Alsheikh Ahmed, Mohanad Ibrahim, Israa Wina, Samah Hassan, Aziza Mohammad Hassan Mohammad Ali, Mohamedzain Elamin, Mukhtar Bastawi, Salih Ahmed, Azza Ballal, Sara Y Mukhtar, Afnan Ali, Rofayda M Mohamad, Alaa Ahmed, Aya Mohamed, Adieb Elgaylani

**Affiliations:** 1 Internal Medicine, The James Cook University Hospital, Middlesbrough, GBR; 2 General Surgery, Al Managil Teaching Hospital, Al Managil, SDN; 3 Surgery, Hamad Medical Corporation, Doha, QAT; 4 Surgery, International University of Africa, Khartoum, SDN; 5 Emergency, Kosti Teaching Hospital, Kosti, SDN; 6 Dermatology, Dr. Health Clinics, Riyadh, SAU; 7 General Surgery, University of Bahri, Khartoum, SDN; 8 Medicine, National Ribat University, Khartoum, SDN; 9 Medicine, University of Gezira, Khartoum, SDN; 10 Pediatric Surgery, Royal Manchester Children's Hospital, Manchester, GBR; 11 General and Laparoscopic Surgery, Fakeeh University Hospital, Dubai, ARE; 12 Preventive Medicine, King Salman Armed Forces Hospital, Tabuk, SAU; 13 Medicine, University of Khartoum, Khartoum, SDN; 14 Medicine, University of Medical Sciences and Technology, Khartoum, SDN

**Keywords:** acute kidney injury, acute pancreatitis, aki, hypoalbuminemia, serum albumin

## Abstract

Acute kidney injury (AKI) is a particularly major complication among patients with acute pancreatitis, which markedly increases the length of hospitalization, morbidity, and mortality. Low serum albumin levels have been associated with disease severity and worse outcomes in various critical illnesses. Accordingly, this meta-analysis was done to assess the association between serum albumin level and AKI in patients with acute pancreatitis.

A systematic review was conducted according to the Preferred Reporting Items for Systematic Reviews and Meta-Analyses (PRISMA) guidelines. The search was performed in PubMed, Web of Science, and Scopus. Pooled standardized mean differences (SMDs) with 95% confidence intervals (CIs) were calculated to investigate the difference in albumin values between AKI and non-AKI patient groups.

A total of seven studies, including 3,496 patients with acute pancreatitis, were included in the meta-analysis. The results indicate that patients who developed AKI had substantially lower serum albumin concentrations at baseline compared to those who did not develop AKI, with the pooled SMD being -0.55 (95% CI: -0.64 to -0.47; p < 0.001). Assessment of between-study variability demonstrated no significant heterogeneity, and sensitivity analysis confirmed robustness of the results.

This meta-analysis demonstrated that hypoalbuminemia could serve as a reliable biomarker for identifying patients at increased risk of kidney injury during the course of acute pancreatitis.

## Introduction and background

Acute pancreatitis is an inflammatory condition of the exocrine pancreas marked by severe abdominal pain, elevated serum amylase and lipase, and potentially organ dysfunction [1,2]. Most cases are mild, but severe cases can lead to complications like pancreatic necrosis and multi-organ failure [1,2]. Some cases develop a severe course of the disease, with mortality rates ranging up to 50% [3]. Therefore, early identification of patients at high risk of poor outcomes is a key clinical challenge.

The disease has a broad clinical spectrum ranging from mild, self-limiting disease to severe systemic inflammation and multiorgan failure. Among its complications, acute kidney injury (AKI) is a serious event that increases morbidity and mortality [4]. Given that, early recognition of AKI in patients with acute pancreatitis is important. The causes of AKI as a result of severe acute pancreatitis include discharge of pancreatic amylase by the injured pancreas with disturbance of renal microcirculation, hypoxemia, and reduction in renal perfusion pressure, intra-abdominal hypertension or hypovolemia, and endotoxins and reactive oxygen species [5].

In the context of severe inflammatory conditions, hypoalbuminemia often develops early due to increased vascular permeability and reduced hepatic synthesis. Low serum albumin levels have been associated with disease severity and worse outcomes in various critical illnesses [6]. Serum albumin, a routinely available and low-cost biochemical parameter, plays important roles in modulating oxidative and inflammatory responses [7].

Although individual studies provide valuable preliminary evidence on the association between hypoalbuminemia and AKI in pancreatitis, they are often limited by sample size and single-center biases. A meta-analysis would establish the strength of the association between hypoalbuminemia and AKI in pancreatitis. In light of this, we are conducting a systematic review of the studies that aimed to investigate the association between serum albumin levels and AKI in patients admitted with acute pancreatitis. By doing so, we aim to justify the benefit of using serum albumin levels in early prognostic assessment and to contribute to the evidence-based clinical practice regarding biomarker-guided management in acute pancreatitis.

## Review

Methods

Search and Screening

This systematic review was conducted according to the Preferred Reporting Items for Systematic Reviews and Meta-Analyses (PRISMA) guidelines [8], with the protocol prospectively registered in the PROSPERO database (CRD420251134478). We searched PubMed, Web of Science, and Scopus from their inception to the end of August 2025 using a strategy combining controlled vocabulary and free-text terms for “acute pancreatitis,” “albumin,” and “acute kidney injury” (see Appendices). References of eligible articles and relevant reviews were screened manually to identify additional studies, with disagreements resolved by discussion and consensus.

Inclusion and Exclusion Criteria

Observational cross-sectional, case-control, and cohort studies that explicitly reported the necessary data on the association between serum albumin levels and AKI were included. The primary outcome was the occurrence of AKI during hospitalization according to consensus criteria or equivalent clinical definitions applied in each study. We left out abstracts, letters, reviews, case reports, articles not in English, and studies lacking adequate data of interest. The initial screening involved reviewing the titles and abstracts of all identified articles to pinpoint relevant ones. Subsequently, a thorough full-text examination was conducted following the inclusion criteria.

Quality Assessment and Data Extraction

The methodological quality of each study was assessed using the Joanna Briggs Institute (JBI) critical appraisal checklists (http://jbi.global/critical-appraisal-tools). Data were extracted using a standardized form that captured study characteristics (first author, year, country, design, sample size), patient demographics, definitions of hypoalbuminemia, AKI diagnostic criteria, serum albumin values in AKI and non-AKI groups. When multiple estimates were reported, those adjusted for the most confounders were selected. For studies reporting medians and interquartile ranges (IQRs), means and standard deviations (SDs) were estimated using established transformation methods by Wan et al. [9].

Data Analysis

Statistical analyses were carried out by determining the pooled standardized mean difference (SMD) estimates with 95% confidence intervals (CIs) to evaluate relationships with serum albumin and AKI. To address the variability among studies, a random effect model (DerSimonian-Laird) was applied. The I^2^ statistical test was utilized to assess heterogeneity across studies, measuring the percentage of variation in effect estimates as a result of heterogeneity instead of random chance. Begg’s and Egger’s tests were applied to estimate publication bias. The trim-and-fill technique by Duval and Tweedie was applied to address the possibility of missing studies in the presence of publication bias [10-12].

Meta-regression analyses were performed to examine heterogeneity and evaluate how particular study characteristics relate to the effect size. Furthermore, leave-one-out sensitivity analyses were performed to assess the robustness of the meta-analyses by examining the impact of individual studies on the results. The significance level for all tests was established at 0.05. The analyses were performed utilizing the meta package in R version 4.4.3 (R Foundation for Statistical Computing, Vienna, Austria).

Results

Study Characteristics

**Figure 1:**
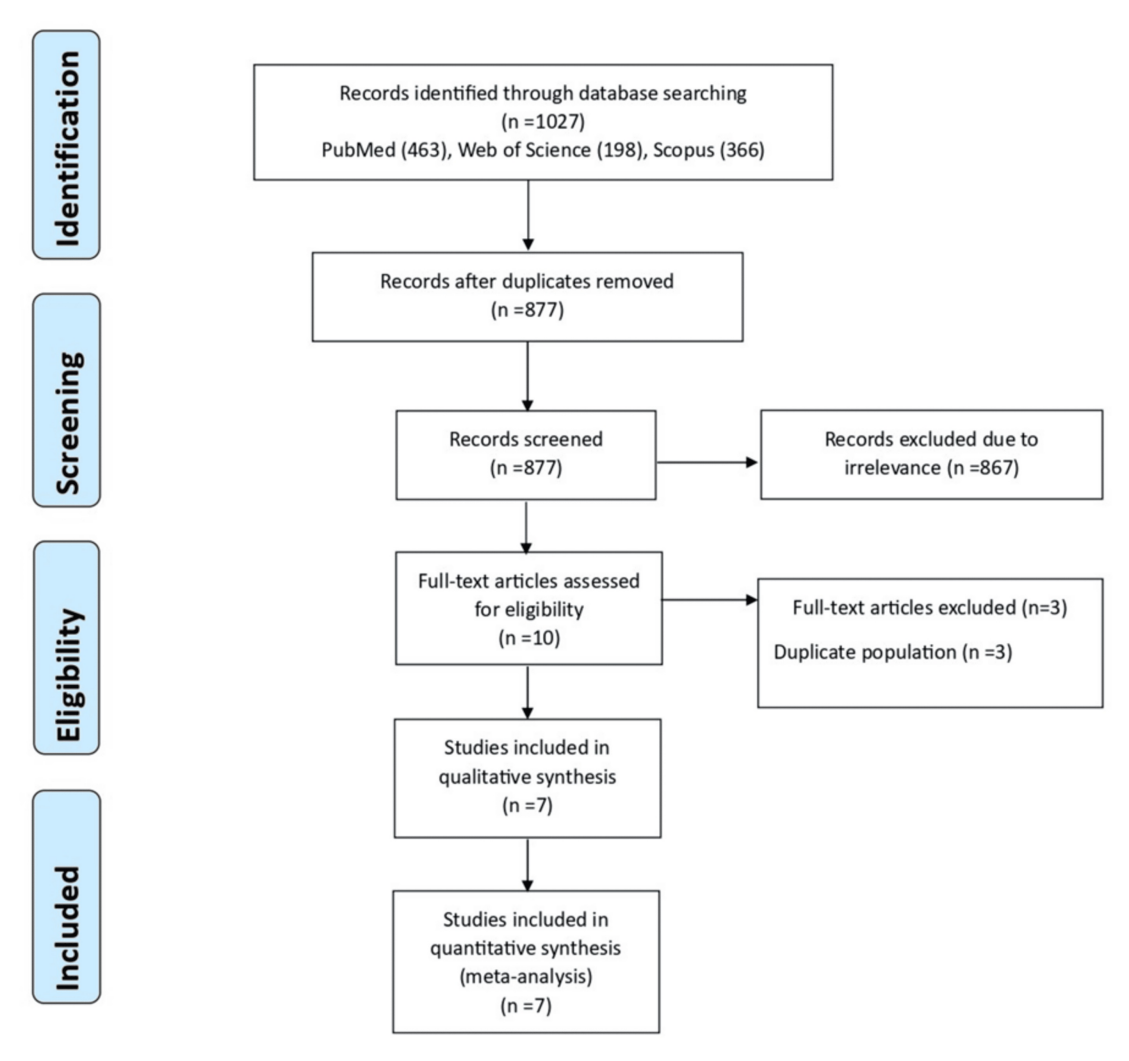
Flow diagram illustrating the study selection procedure.

The graphic flow of the study's selection process is demonstrated in Figure 1. The first electronic database search produced a total of 1,027 records. After eliminating duplicate entries, 877 studies were left. These studies went through a process of screening titles and abstracts. At this phase, 867 studies were eliminated because they were not relevant, and the complete texts of the other records were examined (Figure 1).

This meta-analysis included seven studies that consisted of 3,496 patients suffering from acute pancreatitis [13-19]. Among the participants, 1,074 patients developed AKI, while 2,422 patients did not experience AKI and were used as controls. The studies were published between 2018 and 2025, including both Asian and European populations. All of the included studies assessed the adult population. The main features of the selected studies, as well as risk of bias assessment, are presented in Table 1.

**Table 1:** Baseline characteristics of the included studies.

Study	Year	Country	No. of patients	No. of patients with severe AP	Mean age (years)	Male (%)	Quality assessment
Biyik et al. [13]	2025	Turkey	210	20	57 ± 19	45.7	9/11
Chai et al. [14]	2018	China	237	12	58.81 ± 15.46	54.43	8/11
Sporek et al. [15]	2016	Poland	65	5	62.2 ± 16	52.3	9/11
Lu et al. [16]	2021	China	218	218	51.1 ± 15.5	49.5	10/11
Uğurlu et al. [17]	2022	Turkey	582	NA	57.9 ±2 1.05	41.9	8/11
Wang et al. [18]	2025	China	714	NA	20.15 ± 5.64	57.56	8/11
Wu et al. [19]	2025	China	1470	166	53.0 ± 17.8	53.7	9/11

Serum albumin concentrations were consistently assessed as a biochemical parameter or laboratory indicator at the time of hospital admission or within the first 24-48 hours of admission across the studies. All of the included studies defined AKI according to the established clinical criteria. Specifically, AKI was diagnosed according to the Kidney Disease: Improving Global Outcomes (KDIGO) guideline, which states that AKI is diagnosed when serum creatinine rises by ≥26.5 µmol/L within 48 hours, or by ≥1.5 times within seven days, or when urine output is <0.5 ml/kg/h for six hours.

Meta-Analysis Results

**Figure 2:**
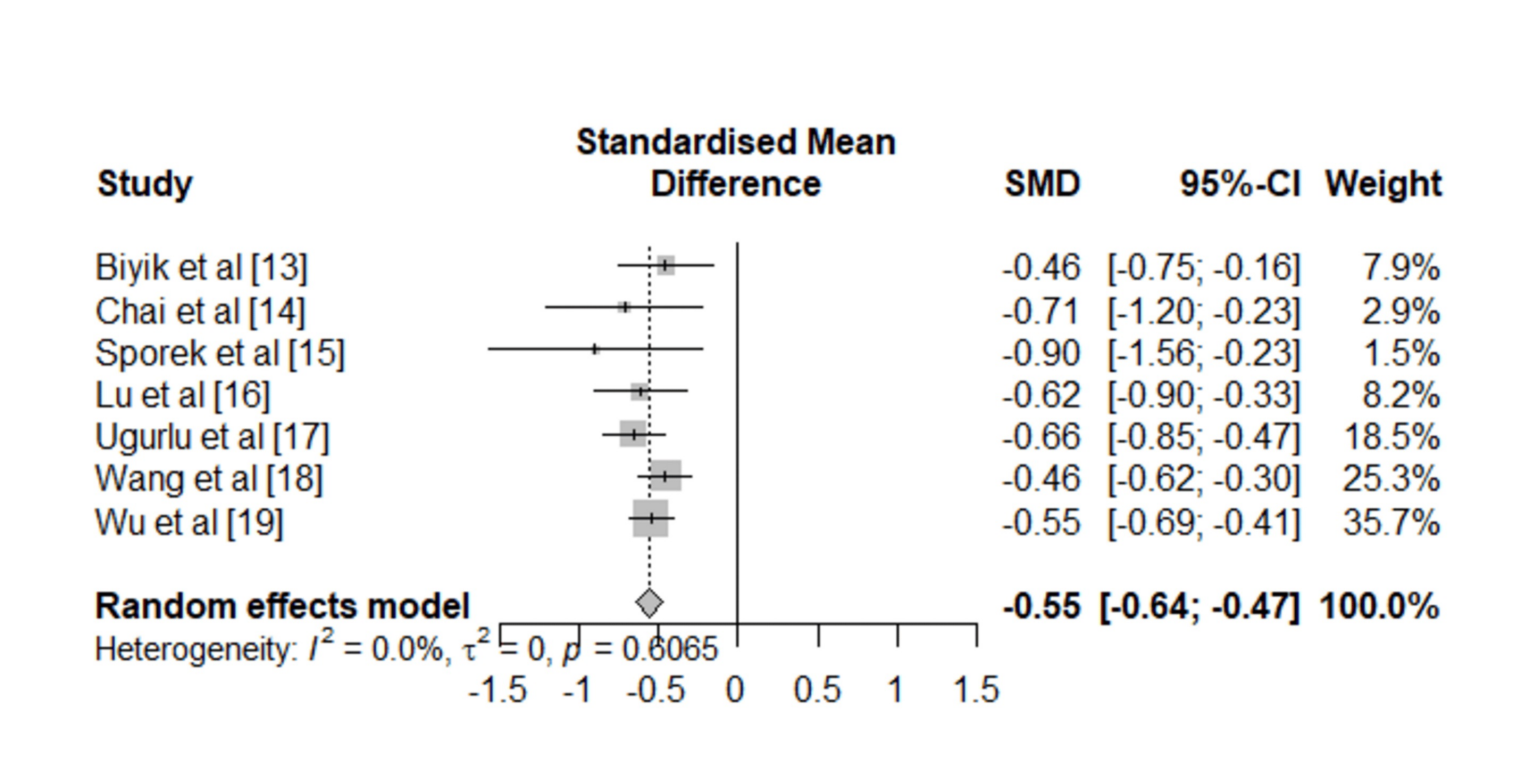
Forest plot displaying the relationship between AKI and serum albumin levels in individuals with acute pancreatitis. AKI: acute kidney injury; SMD: standardized mean differences; CI: confidence interval References [13-19]

The quantitative synthesis indicates that patients who developed AKI had substantially lower serum albumin concentrations at baseline compared to those who did not develop AKI. Using a random-effects model, the pooled SMD was -0.55 (95% CI: -0.64 to -0.47; p < 0.001) (Figure 2).

**Figure 3:**
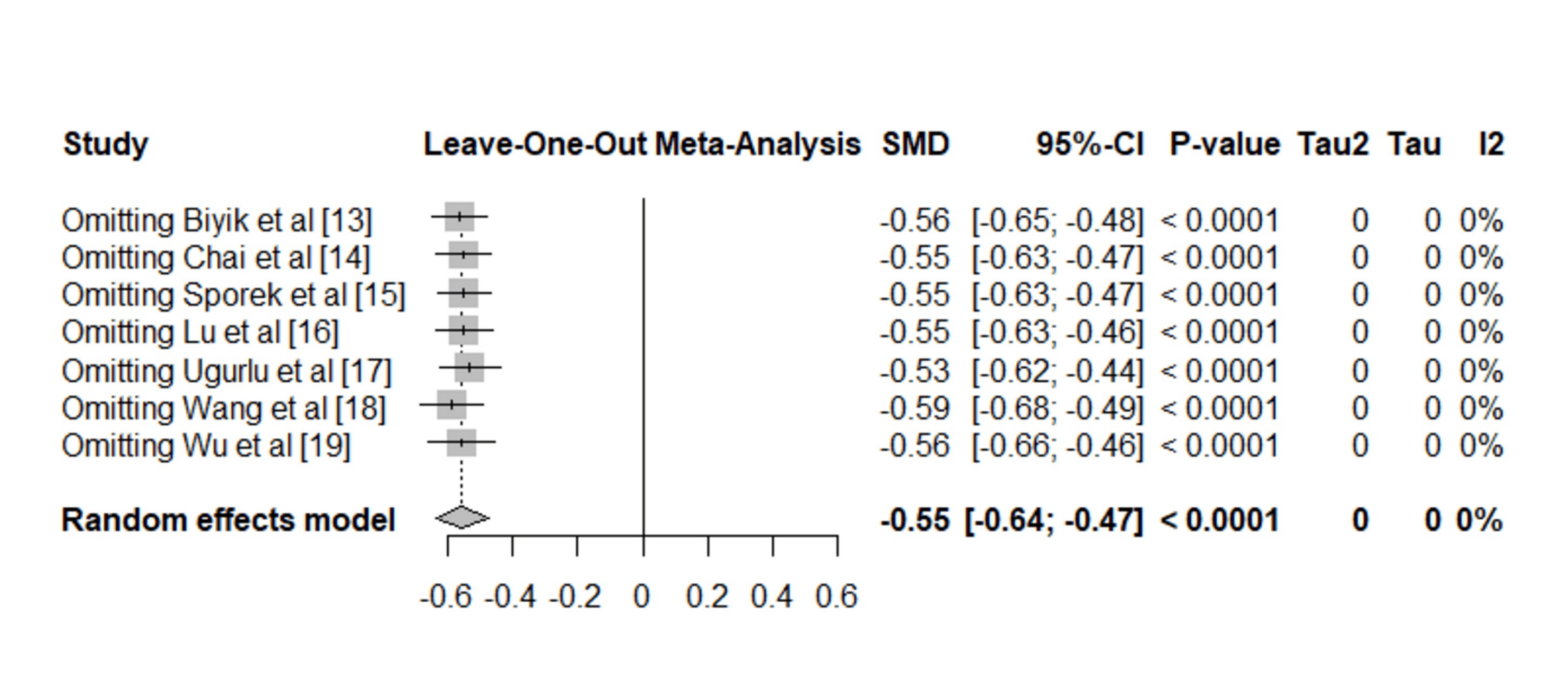
Sensitivity analysis of the association between serum albumin and AKI. AKI: acute kidney injury; SMD: standardized mean differences; CI: confidence interval References [13-19]

Assessment of between-study variability demonstrated no significant heterogeneity (I^2^ = 0.0%, τ^2^ = 0). Cochran’s Q statistic was also non-significant (Q = 4.52, p = 0.61), further supporting the consistency of findings across different studies. To evaluate the stability of the results, a leave-one-out sensitivity analysis was performed. Exclusion of each individual study in turn did not materially alter the pooled effect size, with SMD estimates consistently ranging between -0.53 and -0.59, all of which remained highly significant (Figure 3). These findings confirm that the association between lower albumin and AKI was not driven by any single study.

**Figure 4:**
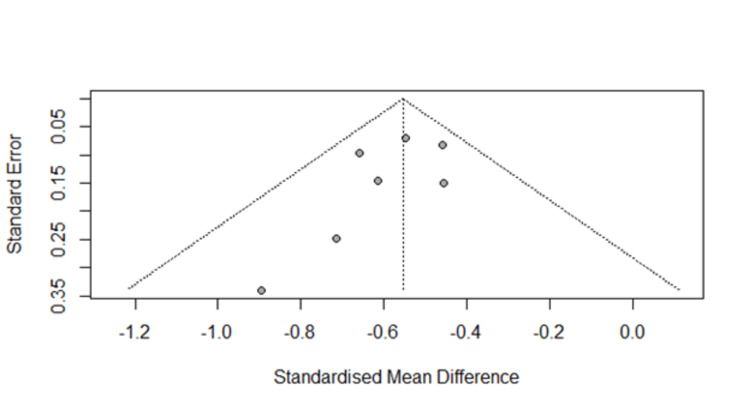
Funnel plot for the meta-analysis of studies on association between serum albumin and AKI. AKI: acute kidney injury

Potential publication bias was assessed using Egger’s linear regression test, which did not reveal evidence of small-study effects (p = 0.23). In addition, visual inspection of the funnel plot demonstrated a largely symmetric distribution of effect sizes around the pooled estimate, further supporting the absence of substantial publication bias (Figure 4).

Meta-regression analyses were conducted to analyze the effect of various variables on the association between serum albumin and AKI. The results showed that none of the following variables had a moderating effect on the outcome; number of participants (coefficient = 0.001, P = 0.684), mean age (coefficient = -0.004, P = 0.148), male ratio (coefficient = 0.009, P = 0.223), percentage of patients with severe acute pancreatitis (coefficient = -0.001, P = 0.722).

Discussion

This meta-analysis found that those who developed AKI had significantly lower serum albumin levels at hospital admission than those who did not. The results were highly consistent across studies, with no significant heterogeneity or publication bias detected. Sensitivity analyses confirmed the stability of the findings, and meta-regression showed that demographic and disease-related factors did not significantly modify the observed association.

These findings reinforce growing evidence that hypoalbuminemia is a reliable predictor of adverse outcomes in acute pancreatitis, including multi-organ failure and mortality [20]. Serum albumin serves not only as a marker of nutritional and inflammatory status but also as an independent prognostic indicator in critically ill patients [6-7,20]. The consistent association observed across studies supports the concept that hypoalbuminemia reflects systemic inflammation, endothelial dysfunction, and early organ impairment. All of which contribute to the pathophysiology of AKI in acute pancreatitis.

Several mechanisms may explain why low serum albumin levels predispose patients with acute pancreatitis to kidney injury. Albumin is essential for maintaining plasma oncotic pressure, and its reduction can lead to interstitial edema, decreased effective circulating volume, and impaired renal perfusion [6,7]. Furthermore, albumin possesses antioxidant, anti-inflammatory, and endothelial-protective properties that help mitigate oxidative stress and vascular injury. In acute pancreatitis, systemic inflammation and increased capillary permeability drive albumin leakage from the intravascular to the interstitial space, further lowering plasma concentrations. Low albumin may also alter drug pharmacokinetics, increasing exposure to nephrotoxic agents and increasing renal risk. These pathways highlight the role of albumin in preserving renal and systemic homeostasis during severe inflammatory stress.

From a clinical perspective, the results suggest that early measurement of serum albumin on admission provides valuable prognostic information. Identifying hypoalbuminemia early could help clinicians recognize patients at increased risk of AKI, enabling more attentive renal monitoring, cautious use of nephrotoxic medications, and tailored fluid management strategies. Although the therapeutic role of albumin replacement remains uncertain, its potential to restore oncotic balance and reduce inflammatory damage merits further exploration [20].

Future research should focus on confirming these associations in large, well-designed prospective studies and on evaluating whether correcting hypoalbuminemia through albumin supplementation can improve renal or overall clinical outcomes in this patient population. Mechanistic studies examining the interplay between albumin, endothelial integrity, and renal microcirculation could yield valuable insights into potential therapeutic targets. Moreover, combining serum albumin with other biomarkers may also enhance their predictive accuracy for early AKI prediction in acute pancreatitis.

This study has strengths that enhance the reliability and generalizability of the results, including strict adherence to systematic review and meta-analysis standards and the absence of statistical heterogeneity or publication bias. However, some limitations should be acknowledged. The included studies were observational, which limits causal inference. In addition, potential confounders such as nutritional status, fluid management, and comorbidities could not be adjusted for. Exclusion of non-English articles could limit representativeness. Despite these limitations, the consistency and robustness of the pooled findings strongly support the association between low serum albumin and the development of AKI in acute pancreatitis.

## Conclusions

This meta-analysis demonstrated that reduced serum albumin levels at hospital admission are significantly associated with a higher risk of AKI among patients with acute pancreatitis. The association was consistent across studies, with no evidence of heterogeneity or publication bias. Due to its accessibility and cost-effectiveness, prognostic value, serum albumin should be routinely assessed as part of the initial evaluation. These results suggest that hypoalbuminemia could serve as a reliable biomarker for identifying patients at increased risk of kidney injury during the course of acute pancreatitis.

## Appendices

**Table 2:** Search strategy.

Database	Search terms
Pubmed	(("Pancreatitis" [tw] OR ""pancreatic"[tw]) AND ((" hypoalbuminemia "[tw] OR " albumin "[tw] OR " serum albumin"[tw] OR " Alb"[tw]) AND ("Acute Kidney Injury"[tw] OR "AKI"[tw] OR "kidney injury"[tw] OR "kidney disease"[tw] OR "renal injury" [tw]OR "renal disease"[tw] OR "renal impairment"[tw]))
Web of Science	(TS= ("Pancreatitis" OR "pancreatic") AND TS= ("hypoalbuminemia" OR "albumin" OR "serum albumin" OR "Alb") AND TS= ("Acute Kidney Injury" OR "AKI" OR "kidney injury" OR "kidney disease" OR "renal injury" OR "renal disease" OR "renal impairment"))
Scopus	(TITLE-ABS-KEY ("Pancreatitis" OR "pancreatic") AND TITLE-ABS-KEY ("Pancreatitis" OR "pancreatic") AND TITLE-ABS-KEY (Acute Kidney Injury" OR "AKI" OR "kidney injury" OR "kidney disease" OR "renal injury" OR "renal disease" OR "renal impairment"))
